# Diagnosis of Kikuchi-Fujimoto Disease: A Comparison between Open Biopsy and Minimally Invasive Ultrasound-Guided Core Biopsy

**DOI:** 10.1371/journal.pone.0095886

**Published:** 2014-05-02

**Authors:** Shan-Chi Yu, Chun-Nan Chen, Hsin-I Huang, Tseng-Cheng Chen, Cheng-Ping Wang, Pei-Jen Lou, Jenq-Yuh Ko, Tzu-Yu Hsiao, Tsung-Lin Yang

**Affiliations:** 1 Department of Otolaryngology, National Taiwan University Hospital and National Taiwan University College of Medicine, Taipei, Taiwan; 2 Department of Pathology, National Taiwan University Hospital, Taiwan; 3 Graduate Institute of Clinical Medicine, National Taiwan University College of Medicine, Taipei, Taiwan; 4 Research Center for Developmental Biology and Regenerative Medicine, National Taiwan University, Taipei, Taiwan; Health Canada and University of Ottawa, Canada

## Abstract

Kikuchi-Fujimoto disease (KFD) is a self-limited disease without any need of surgical treatments. Sampling of tissue is the only invasive procedure during the clinical course. However, the standard sampling procedure with accuracy, minimal invasiveness, and esthetic maintenance has not been established yet. In this study, a retrospective review of clinical utility and pathological presentations of the ultrasound-guided core biopsy (USCB) and the open biopsy (OB) in consecutive KFD patients. From 2010 to 2012, 34 consecutive patients were enrolled. USCB was performed in 11 patients, and OB was done in 26 patients. KFD was confirmed in 82% cases by USCB. Similar pathological presentations were found both in the specimens of USCB and OB. In the three patients who had received both USCB and OB, KFD was confirmed by USCB in one case, while two by OB. Sampling errors were found both in USCB and OB. For diagnosing KFD, USCB can serve as the first-line diagnostic tool. OB can be applied only in the failed cases of USCB.

## Introduction

Kikuchi-Fujimoto disease (KFD), also known as histiocytic necrotizing lymphadenitis, is a reactive disease. Although the pathogenesis remains unknown, the clinical and pathological features of KFD had been described in several series [1]–[3]. Cervical lymphadenopathy and fever are the most common clinical presentations [4], [5]. Skin rash, arthritis, hepatosplenomegaly, leukopenia, elevated erythrocyte sediment rate, and anemia are also noted [6].

KFD has a self-limited clinical course. Most KFD patients recover without any treatments. Therefore, a biopsy for tissue proof is the most invasive procedure during clinical courses. The open biopsy (OB) is routinely used as a standard method to harvest specimens. However, OB is invasive, and sometimes needs general anesthesia and hospital care [7]. Scars are left on the neck, which deteriorate cosmetic outcomes. Because of these clinical drawbacks, to develop an alternative sampling method with minimal invasiveness is required. Surgical interventions are not necessary if the specimens are sufficient to confirm the diagnosis.

Some methods with minimal invasiveness have been clinically used for tissue sampling of cervical diseases. Fine needle aspiration (FNA) is a well-established procedure. However, FNA is limited in some cervical diseases because inadequate specimens and false-negative cytological readings are frequently encountered [7], [8]. Ultrasound-guided core biopsy (USCB) is another sampling method of cervical diseases. In contrast to cytological findings provided by FNA only, USCB can harvest specimens for a standard examination of histopathology and immunohistochemistry. Recently, a growing number of studies demonstrate clinical utility of USCB in diagnosing cervical diseases [9]–[11]. It is possible for USCB to achieve a higher specificity in detecting malignancy, and a higher sensitivity in confirming lymphoma [12]. Accordingly, USCB is a promising alternative to OB for cervical diseases [13], [14].

Although USCB has been used for cervical lymphadenopathy, its role of diagnosing KFD has seldom been addressed. In this study, we aimed to evaluate the clinical utility and pathological findings of USCB and OB in diagnosing KFD. The report provides useful information of selecting the appropriate sampling approach of KFD.

## Materials and Methods

### Patients

From 2010 to 2012, the consecutive patients who were suspicious of having KFD by presenting clinical symptoms and signs were enrolled. This study had been approved by the Research Ethnic Committee of National Taiwan University Hospital. The clinical and laboratory information of all enrolled patients were reviewed.

### Procedure of USCB

**Figure 1:**
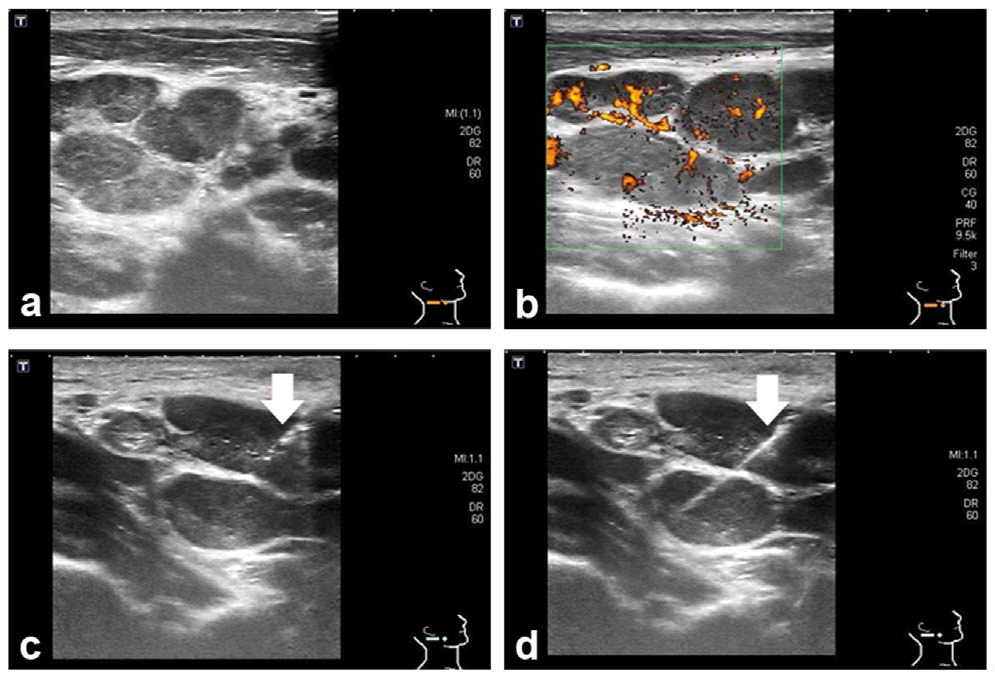
Sonographic presentation and USCB of KFD. a. Multiple lymph nodes with heterogeneous echogenicity and well-defined margins. b. Color duplex mode showed central lymphatic hilus in the enlarged nodes. c. A core needle (arrow) was inserted into the lymph nodes for tissue harvest. d. The inner needle (arrow) was pushed forward to expose the notch for harvesting tissue specimens within the lymph nodes.

The patient was placed in a supine position with the neck hyper-extended. Each patient signed informed consents. The neck skin was disinfected and draped in the standard manner of antiseptic procedure. An ultrasound examination was performed with a 12 MHz linear probe (Toshiba Aplio SSA790 diagnostic ultrasound system, Tochigi-ken, Japan). For USCB, sonographic features and the location of targeted lymph nodes were evaluated and recorded. A color-duplex model was used to avoid vascular injury during USCB. After identifying the safest path to the targeted lesions, local anesthesia was applied. An 18-gauge biopsy needle (Temno Evolution Biopsy Devices, Cardinal Health Inc., Dublin, USA) was guided by ultrasound for sampling. After tissue harvesting, the specimen was removed from the needle notch, checked for quality and quantity, and fixed in formalin. One tissue sample was first collected. If the tissue quality and quantity was not good enough in the gross examination, the second shot was performed. Specimens harvested from cervical lesions were sent for pathological diagnosis. After the procedure, oozing from the puncture wound was controlled with pressure for 5 minutes. The patient was observed for 30 minutes. If there were no signs of complications, the patient was then discharged (Fig 1).

### The procedure of open biopsy

An incision was made just above the palpable mass along the skin crease after local anesthesia. Dissection was performed in a subplastymal manner to carefully separate the surrounding soft tissue, which facilitate to expose the lymph nodes. Either excisional or incisional biopsies were performed for harvesting the tissue samples of lymph nodes. The specimens were fixed and sent for pathological examinations. After hemostasis, the wound was closed and cared in a standard manner. If general anesthesia was required for the procedure of OB, especially for the deep-seated lymph nodes, endotracheal intubation was used. After the procedure, the patient was admitted and stayed in hospital for post-operative care.

### Histopathological examination

The slides of all patients retrieved from the archive of Department of Pathology, were evaluated in the standard pathological examinations, including hematoxylin-eosin stain, histochemistry and immunohistochemistry. The findings of KFD were reviewed independently by two independent pathologists who were blind to clinical information. The pathological features were scored semi-quantitatively. The amount of plasmacytoid dendritic cell was evaluated by immunohistochemical stain for CD123 (IL-3RA, 1:50, BD Biosciences, USA).

### Statistical analysis

For statistical analysis, continuous data were compared by Student's t-test, paired t-test, and ANOVA, and categorical data were compared by Fisher's exact test and Chi-square test. Statistical significance was set at *p*<.05. Statistical analysis was performed using GraphPad Prism5.

## Results

### Patients

Thirty-four consecutive patients diagnosed as KFD were enrolled. The diagnoses of all patients were confirmed pathologically by the features compatible with that of Kikuchi's disease. The mean age of all patients was 27 years old. There were 15 males and 19 females. Cervical lymphadenopathy was the most common clinical presentation. Twenty-nine cases had unilateral cervical lymphadenopathy, either solitary or multiple, and the other five patients had bilateral neck lymphadenopathy. Fever was found in 10 patients. Abnormal hemograms were noted in 12 patients with a wide variety of abnormality including anemia, leukocytosis, leukopenia, and mild thrombocytopenia. Elevated serum lactate dehydrogenase, increased C-reactive protein, and positive anti-nuclear antibody were found. The patients were only followed up without any treatment. During the average 12 months follow-ups, no patients had developed systemic lupus erythematosus (SLE) or malignant lymphoma (Table 1).

**Table 1:** Demographic and clinical features of patients.

Parameters	
Patient number	34
Mean age (years old),	27 (8–45)
Sex (Male :Female)	15: 19 (1: 1.27)
Associated disease	
Autoimmune disease	3 (9%)
Malignancy	2 (6%)
Chief complaint	
Neck mass	31 (91%)
Fever	3 (9%)
Mean duration (days)	25 (1–150) days
Extent of lymphadenopathy	
Unilateral, solitary	15 (44%)
Unilateral, multiple	14 (41%)
Bilateral	5 (15%)
Symptoms and signs	
Fever	10 (29%)
Night sweating	1 (3%)
Body weight loss	1 (3%)
URI symptoms	2 (6%)
Laboratory data	
Abnormal complete blood count	12/26 (46%)
Elevated lactate dehydrogenase	18/21 (86%)
Elevated C-reactive protein	2/8 (25%)
Positive anti-nuclear antibody	4/8 (50%)
Positive tissue culture	0/10 (0%)
Follow-up (months)	12 (4–37)

### Methods of sampling

Among all enrolled patients, 11 patients received USCB, and 26 patients received OB. USCB specimens were obtained from neck lymph nodes under ultrasound guidance without any notable complications. Seven patients needed general anesthesia for OB. The average time of OB was 32 minutes while that of USCB was 10 minutes (Table 2).

**Table 2:** Comparison of the specimens harvested by USCB or Open Biopsy.

		USCB	OB	p-value
Number of specimens		11	26	
Age (years)		27.6±9.6 (18–46)	27.1±11.7 (9–49)	NS
Gender				NS
Male		2	13	
Female		9	13	
Anesthesia				<0.05
General		0	10	
Local		11	16	
Procedure time (cm)		10.0±3.2(5–16)	32.4±10.6 (19–65)	<0.05
Hospital stays (days)		0 (0–0)	3.3 (0–5)	<0.05

NS: non-significant.

Of the 11 patients receiving USCB, KFD was diagnosed in 9 patients (82%). Atypical lymphoid infiltrates were found in the other 2 patients and subsequent OB was arranged. Of the 9 patients who had a diagnosis of KFD by USCB, one patient also received OB. In summary, three patients received both USCB and OB. Overall, there were 11 pathological specimens from USCB and 26 from OB.

### Pathological presentation of specimens harvested from open biopsy

**Figure 2:**
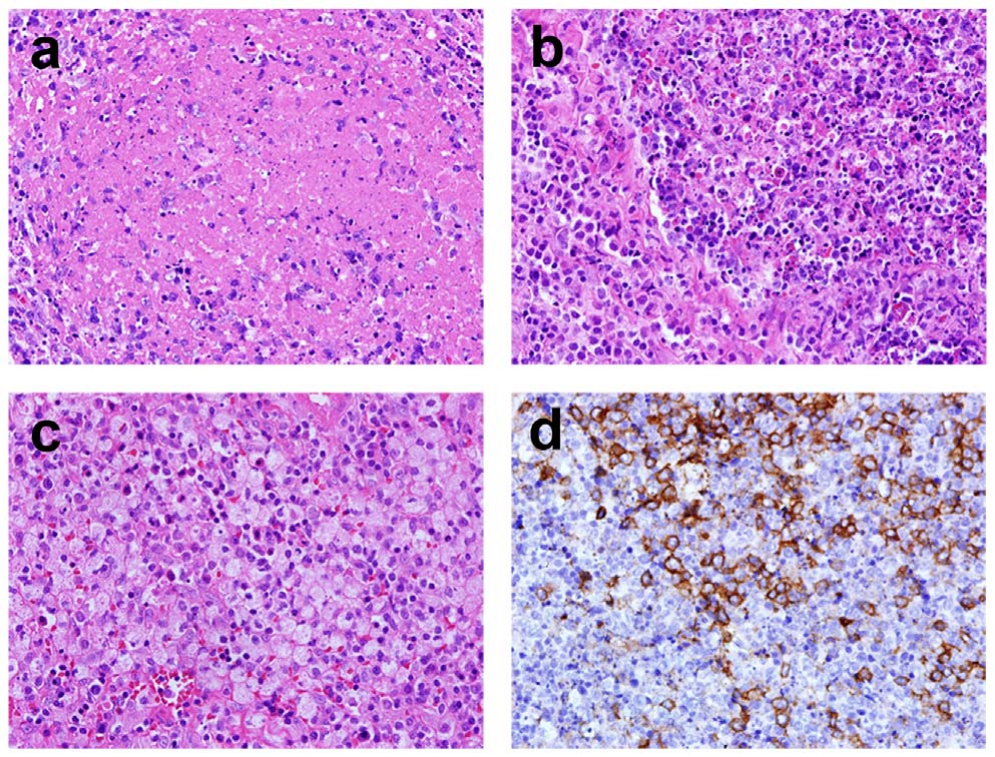
Representative photographs of an open biopsy specimen (400x). a. Necrosis, usually with blurred cell shadow, was mixed with karyorrhexis and histiocytes. b. Numerous histiocytes engulfed karyorrhectic debris. c. Foamy histiocytes were identified. d. Immunohistochemical stain for CD123 highlighted plasmacytoid dendritic cells. Hematoxylin counterstain revealed abundant karyorrhexis around the plasmacytoid dendritic cells.

Twenty-six specimens harvested from OB specimens were analyzed. Necrosis was present in 22 cases (84%), 11 (42%) with large geographic necrosis and 11 (42%) with smaller necrotic foci. The necrosis often has karyorrhexis, histiocytes (Fig 2a) and sometimes immunoblasts. Karyorrhexis was conspicuous in all cases (Fig 2b). Fourteen (54%) had extensive karyorrhexis while 12 (46%) had focal lesions. Histiocyte infiltration was found in all, and foamy histiocyte infiltration was marked in 6 (23%) cases (Fig 2c). Clustered plasmacytoid dendritic cell, which was highlighted by CD123 staining, was marked in 24 (92%) cases (Fig 2d). Small amount of plasma cells were noted in 3 cases (12%). Fibrovascular organization was seen in 2 cases (8%). Pericapsular inflammation was noted in 8 cases (31%). Because of extensive necrosis and histiocyte infiltration, the morphology of follicles varied (Table 3).

**Table 3:** Pathological presentation in the specimens harvested from OB.

Histological features	Case number	Percentage
Karyorrhexis		
Focal	12	46%
Extensive	14	54%
Necrosis		
Absent	4	15%
Small foci	11	42%
Large geographic	11	42%
Histiocyte aggregation		
Mild	17	65%
Marked	9	35%
Crescentic histiocyte	26	100%
Foamy histiocyte aggregation		
Absent	15	58%
Mild	5	19%
Marked	6	23%
Clustered PDC		
Mild	2	8%
Marked	24	92%
Presence of plasma cells	3	12%
Fibrovascular organization	2	8%
Pericapsular inflammation	8	31%
Lymphoid follicle		
Hyperplastic	8	31%
Normal	6	23%
Atrophic	1	4%
Indiscernible	11	42%

PDC: plasmacytoid dendritic cells.

**Figure 3:**
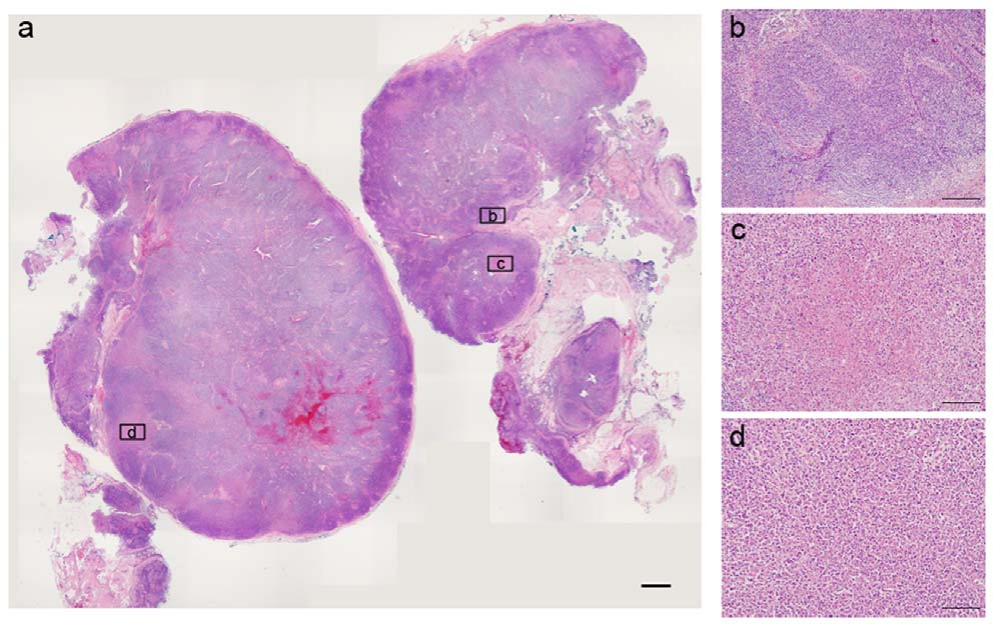
Variable pathological presentations of KFD were shown within one lymph node. The left panel is the scanning view of a bisected lymph node. (Scale bar: 1 mm). The high power view of areas b, c, and d were shown in the right panels (Scale bar: 200 µm). Area b showed nonspecific reactive lymphoid hyperplasia. Area c showed necrosis with karyorrhexis. Area d showed. histiocytic infiltration with karyorrhexis. It demonstrated the possibility of sampling bias if distinct sites of the lesion were harvested.

**Figure 4:**
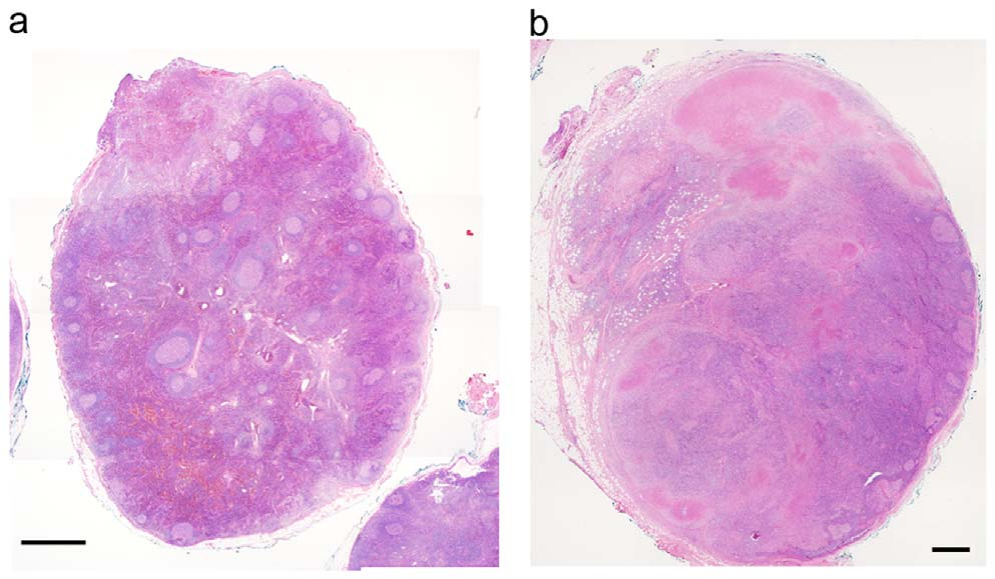
Variable pathological presentations of KFD among different lymph nodes obtained from a surgical biopsy. a. One lymph node showed reactive follicular hyperplasia without evidence of KFD. b. The other lymph node showed specific features of KFD with paracortical necrosis and histiocytic infiltration. (Scale bar: 1 mm).

The heterogeneous presentations of specific KFD pathological characterization were noted either within the same lymph nodes or among different lymph nodes. These typical pathological finding of KFD was either widespread (9 cases, 35%), or limited in focal area (17 cases, 65%) (Fig 3). Among 11 patients with multiple lymph nodes, the morphological variation among lymph nodes was evident in 9 cases (82%) (Fig 4).

### Pathological presentations in the specimens harvested from USCB

**Figure 5:**
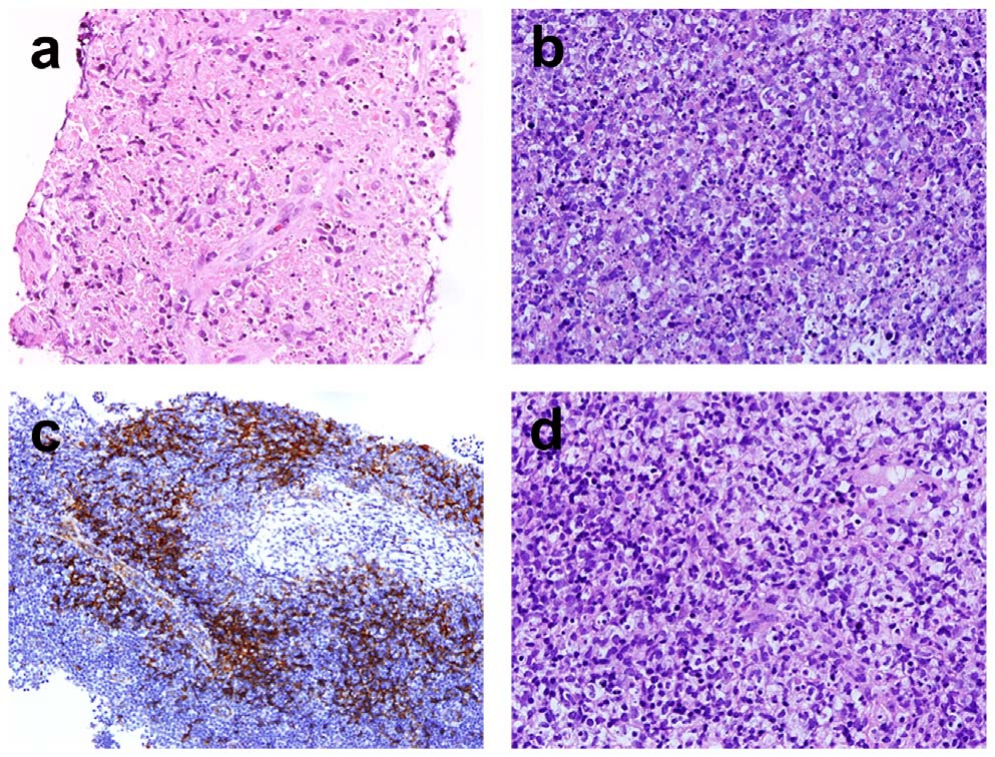
Representative photographs of a USCB specimen. a. Necrosis was mixed with karyorrhexis and histiocytes (H&E, 400x). b. Conspicuous karyorrhexis accompanied by histiocyte aggregation (H&E, 400x). c. Clustered plasmacytoid dendritic cells highlighted by CD123 (200x). d. Increased histiocytes were observed (H&E, 400x).

In addition to the specimens harvested from OB, the pathological findings of those from USCB were reviewed. The pathological presentation of individual USCB specimen was listed in table 4. Diagnosis was made by USCB alone in case 1 to 9. The diagnosis of case 10 and 11 was confirmed by subsequent OB. Necrosis and karyorrhexis were distinctive features of KFD in USCB specimens. Necrosis was mixed with karyorrhexis and histiocytes in 5 cases (45%) (Fig 5a). Karyorrhexis was present in 9 cases (82%), either in necrotic or viable area (Fig 5b). Crescentic histiocytes were noted in 9 cases (82%). Clustered plasmacytoid dendritic cells was present in 7 cases (64%) (Fig 5c). Histiocyte infiltration was found in 11 cases (100%) (Fig 5d). No foamy histiocytes, multinucleate giant cells or granuloma formation was noted. Plasma cells were present in one case (9%).

**Table 4:** Demographic data and pathological presentation in the specimens harvested from USCB.

Case No	Age	Sex	Specimen length (mm)	Necrosis	Karyorrhexis	Histiocyte infiltration	Crescentic histiocyte	Foamy histiocyte	Clustered PDC	Plasma cells
1	30	F	4	-	+	+	++	-	+	-
2	28	F	7	-	++	++	++	-	+	-
3	20	F	9	-	++	++	++	-	+	-
4	18	F	9	-	++	+	++	-	+	+
5	31	F	5	++	++	++	++	-	+	-
6	18	F	3	+	++	+	++	-	-	-
7	45	M	9	+	++	+	+	-	+	-
8	19	M	14	++	++	+	+	-	+	-
9	26	F	8	++	++	+	+	-	-	-
10*	44	F	8	-	-	+	-	-	-	-
11*	25	F	10	-	-	++	-	-	-	-

*The diagnosis of KFD is unable to be made by USCB.

### Comparison of pathological findings of the specimens harvested from open biopsies and USCB

**Figure 6:**
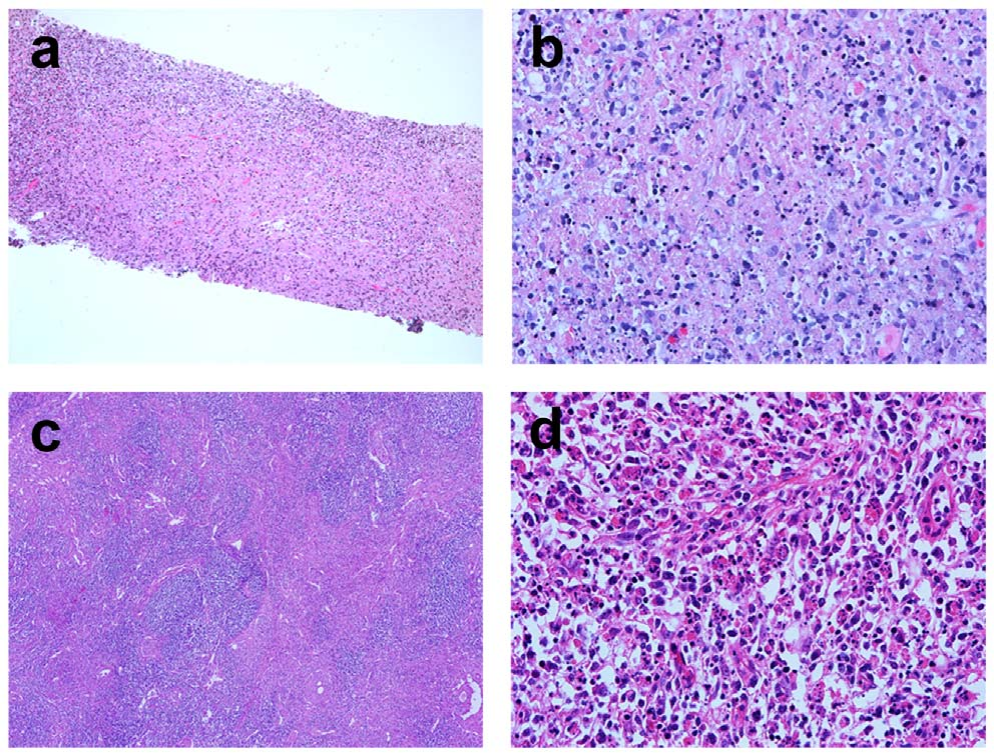
A case of KFD diagnosed by the specimens harvested from USCB instead of OB. a, b. Specimens of USCB. A focus of necrosis mixed with karyorrhexis, histiocytes and immunoblasts (H&E. a, 100x b, 400x). c, d. Specimens of OB. Nonspecific reactive lymphoid hyperplasia with occasional fibrovascular organization (c, 40x). There was only one small focus of aggregated histiocytes accompanied by karyorrhexis (d, x400).

**Figure 7:**
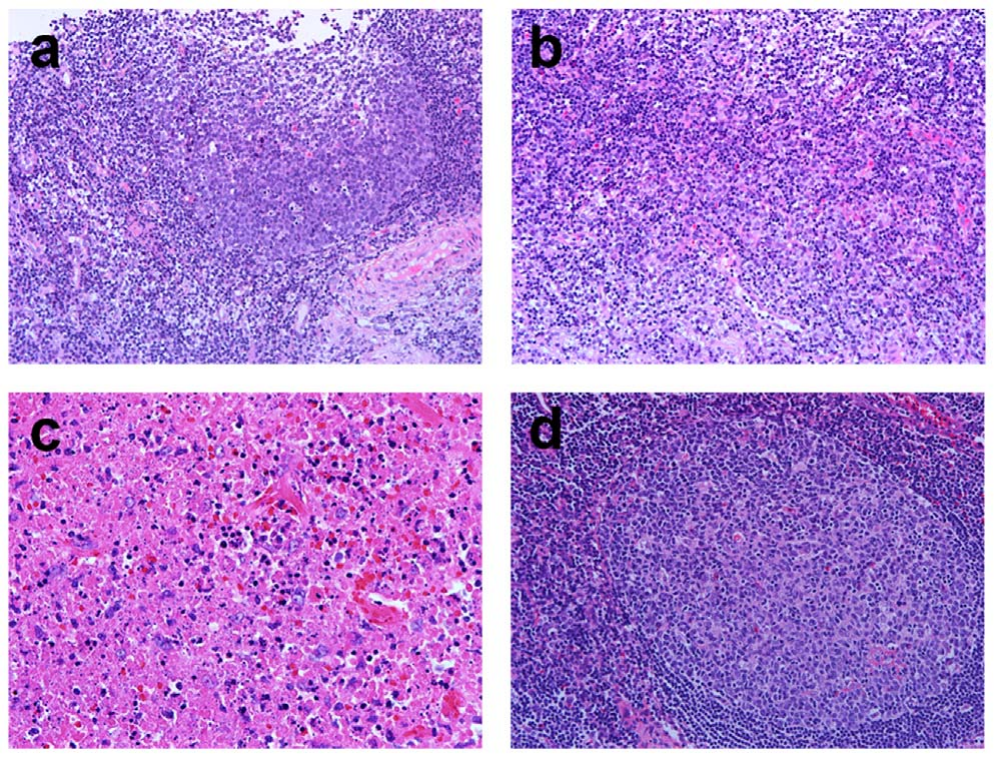
A case of KFD diagnosed by the specimens harvested from OB rather than USCB. a, b. Specimens of USCB. Reactive lymphoid follicles with tingible bodies (a, 200x). Mildly increased histiocytes were noted between follicles, but no karyorrhexis was seen (b, 200x). c, d. Specimens of OB. Distinctive histology with necrosis and karyorrhexis was prominent (c, 400x). Reactive follicular hyperplasia was observed in the viable parts (d, 200x).

In our series, three patients had received both USCB and OB. One case was diagnosed as KFD by USCB, and another two cases were confirmed by OB. In the case diagnosed by USCB, the specimen showed specific features of KFD including evident necrosis, numerous karyorrhexis, and increased immunoblasts (Fig 6a, 6b). Samples taken from OB merely showed nonspecific reactive lymphoid hyperplasia and fibrovascular organization (Fig 6c). There was only one small focus with coagulative necrosis, karyorrhexis, accompanied by plasma cell infiltration (Fig 6d). This case indicated that diagnosis of KFD is sometimes easier to be made by USCB than OB. On the other two cases, the histopathological pictures of USCB specimens showed benign lymphoid tissue with a few lymphoid follicles (Fig 7a). Mild histiocyte infiltration was present, but no karyorrhectic debris was seen (Fig 7b). These pictures favored the diagnosis of reactive lymphoid hyperplasia. In the specimens harvested from OB, there was prominent geographic necrosis and karyorrhexis at paracortex accompanied by histiocytic infiltration. (Fig 7c, d).

## Discussion

In this study, we demonstrated that KFD could be diagnosed either by USCB or OB. The sampling errors may happen both in USCB and OB. When the diagnosis is confirmed by USCB, further OB is unnecessary and the invasive surgical intervention can be avoided.

KFD is a self-limited disease which resolves spontaneously. Confirmation of diagnosis based on tissue sampling is critical. Incisional or excisional biopsies are usually regarded as diagnostic tools of KFD. However, OB is an invasive surgical intervention. It is time-consuming and leaves a visible scar on the neck. General anesthesia, wound care, and hospital stay may be required for OB. For a self-limited disease like KFD, invasive diagnostic procedures are unnecessary if an alternative method is available. To this end, USCB and FNA are alternative choices. A few series of KFD using FNA concluded that FNA is not useful [15], [16]. Most cases suspicious of KFD can be diagnosed by USCB in our series. USCB is an office-based procedure that has the characteristics of minimal invasiveness and preciseness. Most importantly, the pathological information including immunohistochemical staining can be provided by USCB. Using USCB, patient suffering, wound care, and medical cost can be reduced.

The major concern of USCB in diagnosing KFD is accuracy, which is largely dependent on the pathological features presented in the harvested specimens. KFD usually shows paracortical foci of necrosis with abundant karyorrhectic debris and numerous histiocytes [1], [2]. Similar diagnostic features of KFD are also found in the specimens harvested by USCB. Necrosis, usually mixed with karyorrhexis and histiocytes, is observed. The morphology of histiocytes may vary, including crescentic histiocytes and foamy histiocytes, but multinucleate giant cells or epithelioid histiocytes are not seen in KFD. Immunohistochemical stain for CD123 can highlight clustered plasmacytoid dendritic cells, which is beneficial to confirm the diagnosis of KFD in USCB specimens.

It is important to make the differential diagnosis of KFD. Therefore, the advantages and disadvantages of different sampling techniques should be verified based on pathological evidences. KFD may mimic malignant lymphoma by showing abundant immunoblasts. The pictures of extensive karyorrhexis of KFD which are found both in USCB and OB can be used for differentiation. The immunohistochemical results from either USCB or OB are also helpful to distinguish two disease entities. SLE lymphadenopathy has nearly identical histopathological features of KFD [17]. Furthermore, the histopathological features of several infectious diseases such as Yersinia enterolitica and typhoid lymphadenopathy may resemble those of KFD [18], [19]. Bartonella henselae, Epstein Barr virus, cytomegalovirus, Toxoplasma gondii, and syphilis are also associated with necrotizing lymphadenitis [4]. However, their clinical presentations are different from KFD. Tuberculosis is one of the infectious diseases that should be carefully distinguished form KFD because the clinical presentations and radiological findings are similar in both diseases [20]–[23]. Tuberculosis mimics KFD when the specimen is composed of abundant caseous necrosis with few or absent epithelioid cells. In contrast to tuberculosis, KFD has no epithelioid histiocytes, granuloma or multinucleate giant cells. An acid fast stain is suggested for the cases suspicious of tuberculosis. In our study, none of the 9 KFD patients diagnosed by USCB had evidence of lymphoma, SLE or specific infectious diseases. All of them had a benign clinical course during follow-up.

Because KFD exhibits marked variation within a lymph node, or among different lymph nodes, sampling error is inevitable. Three cases of our series demonstrated the possibility of sampling bias, no matter in USCB or OB. In some cases, the typical morphology of KFD is restricted locally, which may not be easily sampled by USCB, leading to inadequate sampling and inaccurate diagnosis (Fig 3). Similar effect is also observed even with OB. Because KFD may exhibit different features among lymph nodes, only few lymph nodes taken form OB may also result in a diagnostic bias (Fig 4). Certainly, more lymph nodes taken for evaluation indeed significantly increase the diagnostic rate of KFD. However, the invasiveness might correspondingly increase, which is not favored for a self-limited, benign disease like KFD. With meticulous histopathological examinations by experienced physicians and pathologists, many KFD cases can be successfully diagnosed by USCB. Therefore, USCB may serve as an alternative sampling method of OB for KFD diagnosis. For routine practices, USCB can provide specimens for pathological examinations and has the advantages of minimal invasiveness, convenience, and low cost. Therefore, USCB can serve as the first-line diagnostic tool of KFD. Multiple shots of USCB are suggested to increase the diagnostic accuracy because of the heterogeneous pathological nature of KFD. An OB can be a backup sampling tool when USCB is futile.

For suspicious KFD patients, USCB can serve as the first-line diagnostic tool. USCB is a sampling procedure with the characteristics of minimal invasiveness and preciseness, and can provide pathological information for diagnosis. Using USCB, the possibility of scar formation, wound care, and medical cost can be reduced.

## Supporting Information

Checklist S1 **A STARD checklist for reporting of diagnostic accuracy in this study.**
(DOC)

Flow Chart S1 **A STARD flow chart shows the process of recruiting patients with suspicious KFD for diagnostic procedures.**
(PDF)
