# Supplementary figures and images for: Diagnosis of Kikuchi-Fujimoto Disease: A Comparison between Open Biopsy and Minimally Invasive Ultrasound-Guided Core Biopsy
Source: PLoS One. 2014 May 2;9(5):e95886. doi: 10.1371/journal.pone.0095886 (PMC4008434; doi:10.1371/journal.pone.0095886)

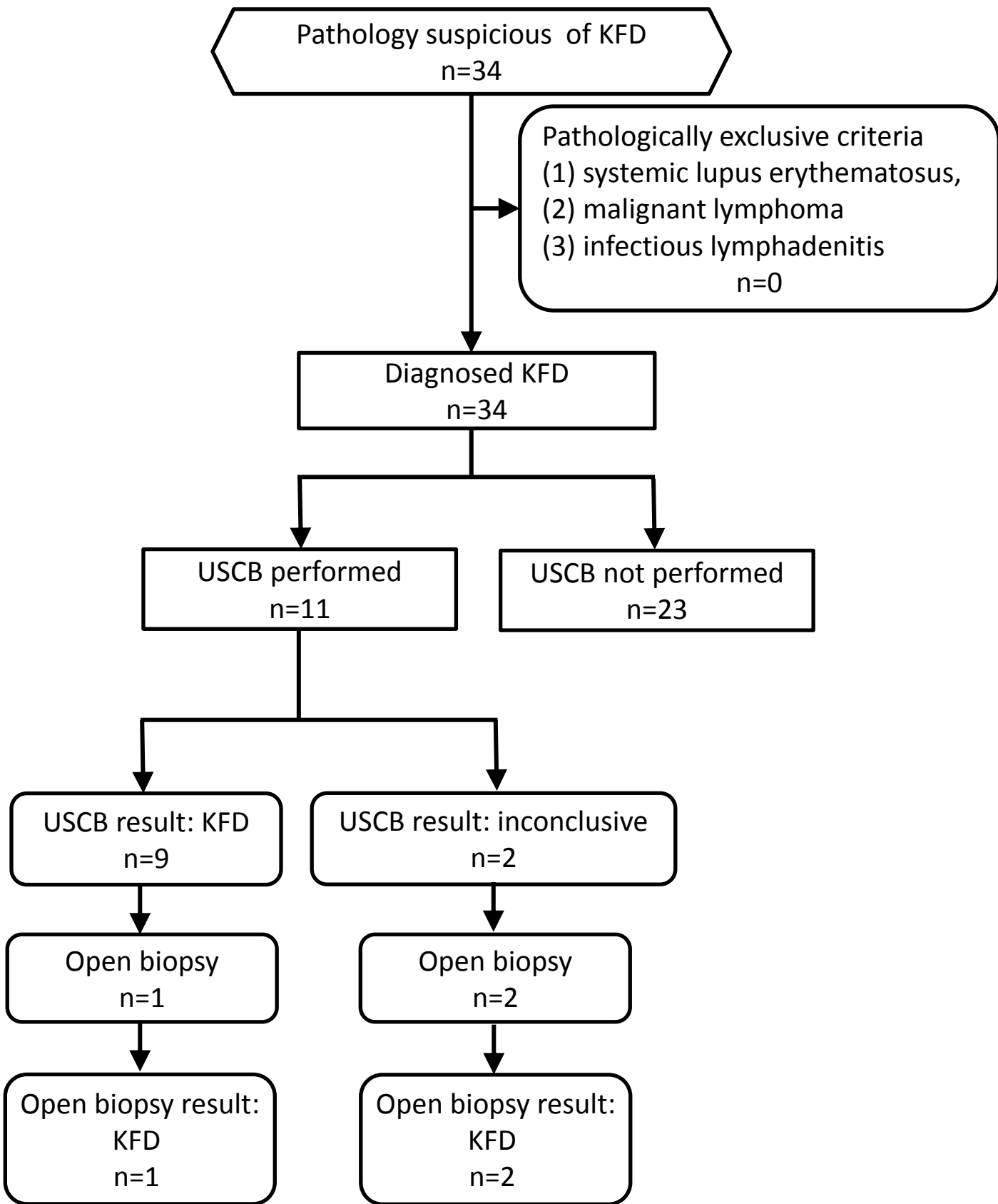

Supplement: Flow Chart S1 — A STARD flow chart shows the process of recruiting patients with suspicious KFD for diagnostic procedures. (PDF) [file pone.0095886.s002.pdf]
